# Hot topics and frontier evolution in college flipped classrooms based on mapping knowledge domains

**DOI:** 10.3389/fpubh.2022.950106

**Published:** 2022-08-16

**Authors:** Liyan Sun, Li Yang, Xue Wang, Junqi Zhu, Xuesen Zhang

**Affiliations:** School of Economics and Management, Anhui University of Science and Technology, Huainan, China

**Keywords:** flipped classroom, mapping knowledge domains, COVID-19, online teaching, CiteSpace

## Abstract

With the outbreak of COVID-19 and the development of online teaching, the online flipping teaching mode has attracted increasing attention. Systematic analysis of the research status and development trend of the flipped classrooms is significant for guiding the improvement of the quality of online flipped teaching. This study used the metrology software CiteSpace to draw a scientific knowledge map of relevant research in the web of science database from 2013 to 2021. It performed visual analysis of research authors, research institutions and countries, keyword clustering, keywords co-occurrence, and keyword time zone distribution. The results showed that: (1) The flipped classrooms research has attracted increasing attention from the social and educational circles, however, the relationship between relevant research authors, institutions, and countries is not close enough, and there is little cooperation. We need to strengthen cooperation further and realize the sharing of high-quality resources; (2) Based on keyword co-occurrence cluster analysis, this study identified three hot topics, namely, preparation before class, classroom activities and consolidation after class; (3) According to the keyword time zone map, this study divided three frontier evolution trends: exploration period, adaptation period, and growth period; (4) Finally, with the spread of novel coronavirus, it is suggested to promote the online flipped classroom teaching mode, and put forward reasonable suggestions from the perspective of teachers, students and researchers, and look forward to the future digital development direction of the flipped classroom.

## Introduction

The worldwide outbreak of COVID-19 has led to major societal changes, including social, economic, psychological, educational, and more (1, 2). The World Health Organization stated this has been a huge threat to human security and social development (3). the field of education, face-to-face courses have been suspended, and schools must adjust teaching activities and enforce virtual learning environments to meet students' learning needs, which forces all teaching activities to change to online forms (4–9). Although online teaching is designed as a supplement to face-to-face instruction, it undertakes all teaching activities in the school (10). In many cases, teachers do not have enough digital skills, and the students' technical environment also has certain limitations highlighting the digital divide's existence (11). Although information and communication technology (ICT) has made progress, educational institutions did not realize the importance of a digital learning environment until the arrival of this pandemic (12). They began to make urgent adjustments to digital resources and educational platforms to make full preparations for online teaching.

However, online teaching continues to face drawbacks, such as fewer learning regulation, fewer interactions and a worse teaching atmosphere; many people find it difficult to provide online education of the same quality as face-to-face classrooms (13). Flipped classrooms refer to the transformation from a teacher-centered teaching mode to student-centered teaching mode, that is to subvert the traditional classroom teaching model. In this teaching model, teachers no longer occupy valuable class time to pass on information, instead, students are allowed to watch the teacher's teaching videos before or after class to learn independently (14). Flipped teaching creates a rich learning environment, breaks the previous learning habits of students, encourages students to study the content of each topic, and performs prominently in autonomy, collaboration, teamwork and exploration (15–17). The flipped classroom model increases the interaction between teachers and students. It can also stimulate students' learning interest and positive attitude and improve students' autonomous learning ability (18). Flipping teaching mode can effectively make up for the shortcomings of online teaching. It can be seen that it is particularly important to carry out online flipping teaching mode actively. The classroom has become a place for students to absorb and consolidate what they have learned. Teachers and students can communicate about the problems in the teaching videos and conduct effective classroom practice (such as cases, experiments, games, simulations, etc.) to gain a deeper understanding. Teachers also help students better master and apply knowledge to improve the usefulness of teaching. A flipped classrooms can take many forms, from being completely face-to-face to fully online (19). Flipped instructions ease the transition from face-to-face to online education (20). Therefore, to effectively deal with the outbreak of COVID-19 and solve the problem of students being unable to attend school, it is necessary to systematically sort out the research status and latest development of flipped classrooms, which is conducive to the perfect combination of online teaching, and a flipped mode, and is of great significance to improve the quality of online education.

In recent years, the flipped classroom teaching model has gradually become the focus of research. Scholars have studied the implementation effect of flipped classrooms in many disciplines, such as public health, nursing, English, medicine, chemistry, economics, psychology, and engineering education. In the public health curriculum, Kang et al. Proved that the flipped classroom, which is based on team learning, improved students' knowledge, problem-solving ability, and learning satisfaction (21). In engineering courses, students had a favorable impression of the flipped classroom teaching model, believing that flipped classroom teaching is helpful for lesson preparation (22). Students believed that there were positive changes in classroom environment perception, flipping preference, responsibility perception, learning motivation, and so on (23). In college English courses, flipped classroom teaching mode can achieve a high resource sharing rate and good teaching effect (24–26). In social sciences courses, active learning made the classroom more attractive, put learning into practice, and made materials more unforgettable (27). In medical courses, the flipped classrooms were found to encourage students to master theoretical knowledge and practical operation ability in clinical practice and improve critical thinking ability and comprehensive quality (28, 29). In computer courses, students' learning motivation has been significantly improved, making the final score less dependent on laboratory attendance (30). In nursing education, flipped classrooms improved the learning outcomes and interest of nursing students (31–34). In programming education, flipped classroom had a positive effect on students' success and self-efficacy, but had no impact on their attitude (35). However, the research results on flipped classrooms are not consistent. For example, Dong et al. stated that flipped classrooms effectively improved students' academic performance and promoted the development of high-level thinking ability but did not affect students' satisfaction and curriculum experience (36). Fakhoury et al. demonstrated that although student examination results did not improve significantly, satisfaction with and participation in course materials improved (37). Pence et al. showed that flipped classrooms could not improve learning motivation and learning strategies (38). Birgili et al. believed that there were problems in the flipped classrooms, such as long teaching video time, poor quality, insufficient lesson preparation, students lacked certain learning motivations, and faced some difficulties in interaction (39). Students believed that the active learning method of flipped classrooms imposes too much workload, and they preferred traditional teaching forms (40). Students choose classroom discussions with experts rather than peers without expertise in this field (41).

Although the views of researchers regarding the flipped classrooms are different, which may be affected by various factors such as limited teaching resources, equipment configuration, technical environment, quality of curriculum design, size, and students' performance (42, 43). In addition, the effect of the flipped classrooms will be effected by students with low abstract ability, students with a poor problem-solving ability, students affected by the digital divide, and teachers' digital skills (44–46). Scholars generally believe that a flipped classroom is more advantageous than a traditional classroom. With the progress of society and the improvement of resources and technology environment, colleges and universities provide more convenient conditions for the implementation of flipped classrooms, which has become the representative of the current teaching mode reform.

The research on flipped classrooms is still in its infancy, the research results are relatively scarce, and a systematic theoretical and practical system has not been formed. Most scholars have conducted comparative studies between flipped and traditional classrooms from the perspective of professional disciplines. Conversely, few have performed a knowledge map analysis of flipped classroom-related literature from bibliometrics. The concept of knowledge mapping originated from a workshop organized by the National Academy of Sciences in 2003 (47). It is a novel method and new field of scientometrics, which reflects the development process and structural relationship of scientific knowledge. It has the dual properties and characteristics of graph and spectrum: it is not only a visualization of a knowledge map but also a serialized knowledge lineage. Knowledge map shows the knowledge unit or knowledge groups between many hidden complex relationships, such as network, structure, interaction, cross, evolution or derivative, etc., these complex knowledge relationships produce new knowledge (47). Therefore, this study conducted a visual analysis of the number of published articles, the authors of literature, research institutions and countries related to the flipped classroom with the help of CiteSpace software to understand the research status of the flipped classroom and then analyze the keywords clustering and keywords time zone, systematically sorting out the theme content and evolution trend of the flipped classroom, hoping to provide some academic reference for the follow-up research in this field and theoretical reference for the effective implementation of the flipped classroom.

## Materials and methods

### Methods

With the development of information visualization, CiteSpace knowledge visualization software has become one of the most popular knowledge mapping tools. CiteSpace is a Java application developed by the Chen Chaomei team of Drexel University (48). It can scientifically measure and visualize literature collections in the fields of natural and social sciences to explore the critical path of the evolution of the discipline and analyze the discipline hotspot and development trends. CiteSpace has been mainly used in psychometrics, computer science, economic management, aviation engineering, library and archives management, information science, and engineering management in recent years. However, there are few research on visual analysis of flipped classrooms in universities by CiteSpace. Since 2012, flipped teaching has become one of the most followed emerging methods in the educational sector (49, 50). To systematically and objectively summarize college research on flipped classrooms and identify hot topics and frontier evolution, this study conducted a visual analysis of the literature from 2013 to 2021 by using CiteSpace.

### Materials

#### Search policies

The Web of Science (WOS) database contains rich and reliable sources of literature data, especially in natural sciences, education, economics, and other social sciences. We took the WOS database as the primary retrieval tool and searched in the Web of Science Core Collection. To capture a wider range of potentially eligible articles, we used the following search terms with Boolean operators: Topic = (“flip^*^” or “invert^*^” or “convert^*^”) and (“class^*^” or “teach^*^”) and (“college^*^” or “university^*^”). The asterisks were used as wildcards to include most common expressions of flipped classroom methods, such as flipped classroom, flipping the classroom, flipped learning, flipped teaching. We searched three groups of search terms separately to form three groups of literature data. Then, we combined the three data groups with “and” in the WOS database. A total of 779 papers were retrieved.

#### Inclusion and exclusion criteria

To analyze the hot topics and frontier evolution of the flipped classroom-related literature, the qualified articles are screened, and their inclusion and exclusion criteria are as follows (Table 1):

**Table 1:** Literature inclusion and exclusion criteria.

**Criteria**	**Indicators**
Inclusion criteria	• Full text of the article is published in English. • Type of publication: article, review, peer-reviewed. • The publication period from 2013 to 2021. [Flipped teaching has become one of the most followed emerging methods in the educational sector since 2012 (49, 50)]. • Study focusing on the flipped classroom pedagogy. • Research about higher education.
Exclusion criteria	• Full text of the article is not published in English. • Type of publication: book, chapters, thesis, meeting abstract, commentaries, protocols, study outlines, government publication, posters, editorial material, duplicates, non-peer-reviewed. • Flipped classroom is mentioned but not the focus of the intervention. • Research not about higher education. • Inadequate description of student performance or learning activities.

#### Data extraction

**Figure 1:**
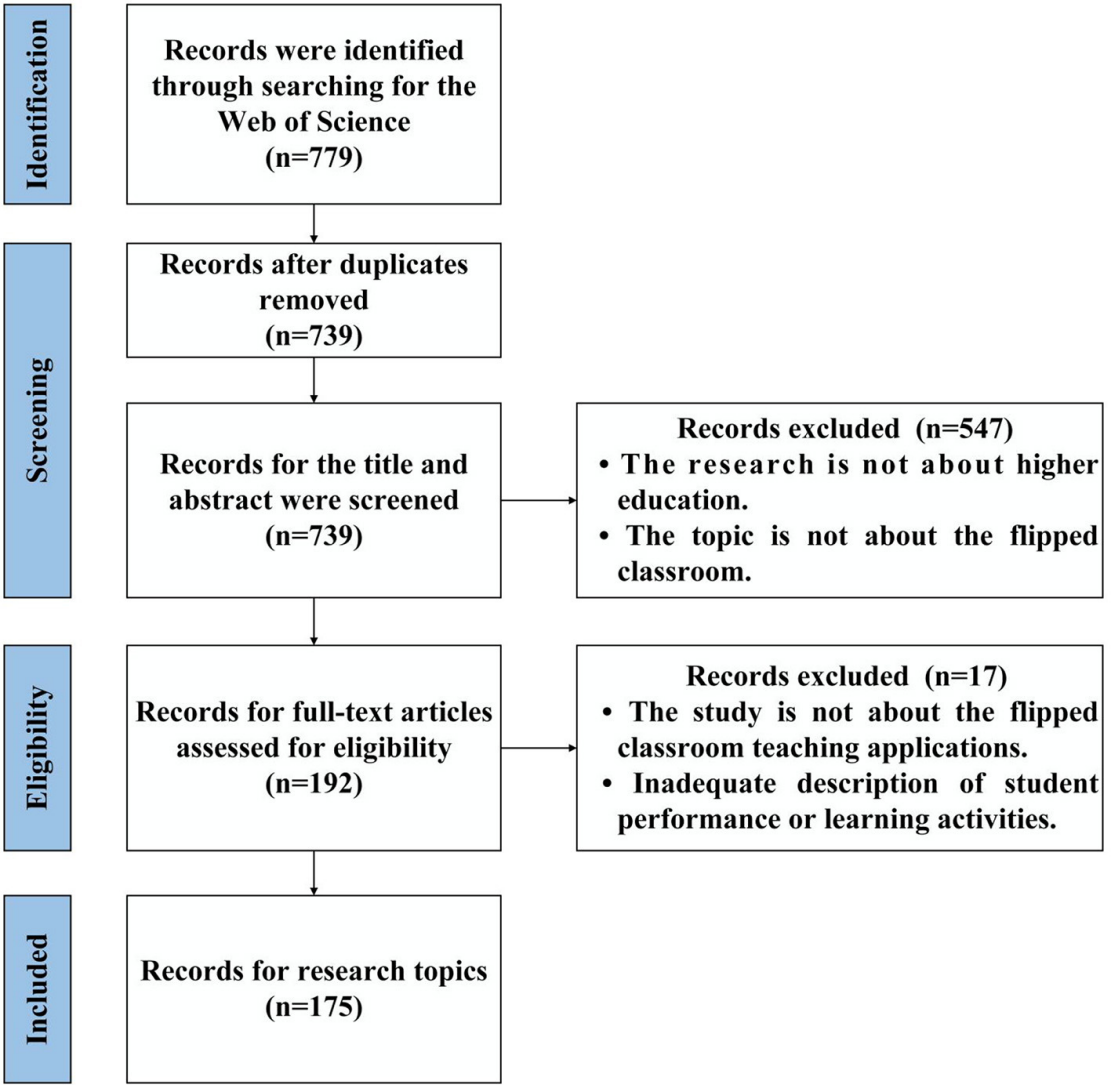
Review literature screening PRISMA flow chart.

The data extraction was carried out by the PRISMA (Preferred Reporting Items for Systematic Reviews and Meta-Analyses) protocols (Additional File). The protocol was registered with the PROSPERO (International prospective register of systematic reviews) (CRD42020194474, 16th October 2020). First, we used the “remove duplicates” function of CiteSpace to delete duplicate literature. Then, the titles and abstracts of the literature were screened according to the inclusion/exclusion criteria, and irrelevant literature were excluded. After reaching a consensus, the full text of the shortlisted studies was downloaded and reviewed. Thirdly, those unrelated studies were excluded according to the full-text review, and all authors assessed their applicability until a consensus was reached. Finally, we finally identified 175 studies. The PRISMA flow chart is shown in Figure 1.

## Results

### Annual number of papers

**Figure 2:**
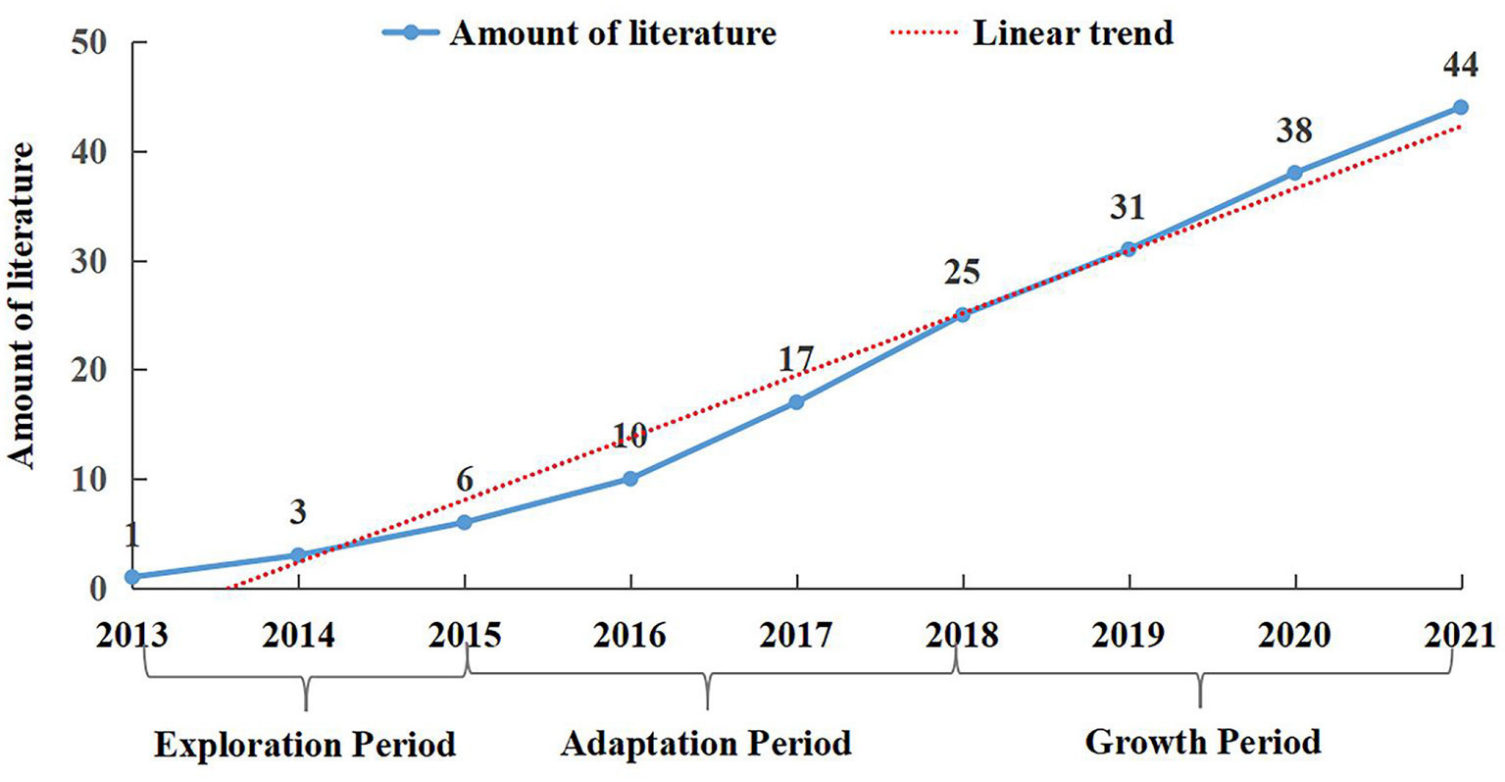
Annual publications of review literature (2013–2021).

The number of papers published on flipped classroom teaching methods annually in international academic journals, to some extent, represents the amount of research in this field, and the change in the number of annual papers reflects the difference in the degree of attention to this field. As shown in Figure 2, from 2013 to 2021, the number of flipped articles published in colleges and universities increased yearly, indicating that flipped classroom in colleges and universities attracted increasing attention. Before 2015, the number of papers published was small, displaying slow growth. This study defines this period as the explore period. From 2016 to 2018, the number of articles published increased significantly, and flipped classrooms attracted more attention. In this period, the progress of information technology might have promoted the development of the flipped classroom, and this period is defined as the adaptation period in this study. From 2019 to 2021, the growth of publications further accelerated, probably because big data and artificial intelligence provide favorable conditions for implementing flipped classrooms, this study defines this period as a growth period.

### Author distribution

**Figure 3:**
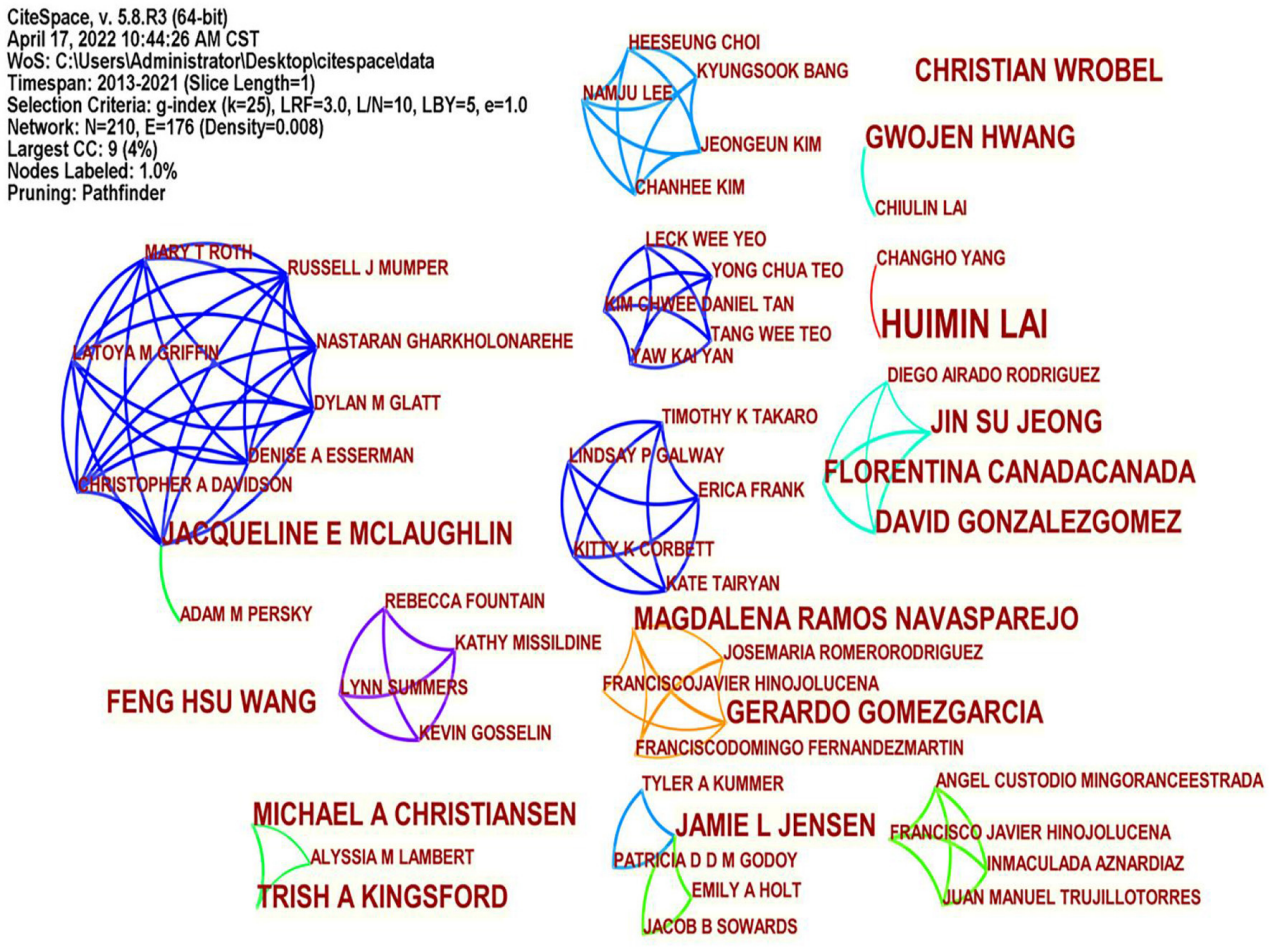
Authors distribution mapping knowledge domains.

Through the analysis of authors who published relevant papers on flipped classrooms, we understand the author's contribution to this field and the cooperative relationship with other authors, which is conducive to promoting the development. Figure 3 shows the cooperation diagram of authors in the flipped classrooms. Two data items, *N* = 210, E = 176, and *N*, represent the node, namely the location node where the author appears in Figure 3. The larger the type, the more frequent the author seems. E represents the line, and the line between the nodes represents the cooperative relationship between the authors. The thicker the line, the higher the frequency of the authors' collaboration. Figure 3 shows that multiple research collaboration teams have been formed, especially those centered around Jacqueline E Mclaughlin, and several scholars have established cooperative relationships. However, the overall level of collaboration among authors is low, and most researchers are independent teams, lacking cross-team cooperation. According to CiteSpace statistics, Huimin Lai ranked first with three publications, two published by some authors and one published by all other authors. Flipped classroom in universities needs to be further studied and promoted.

### Institutions distribution

**Figure 4:**
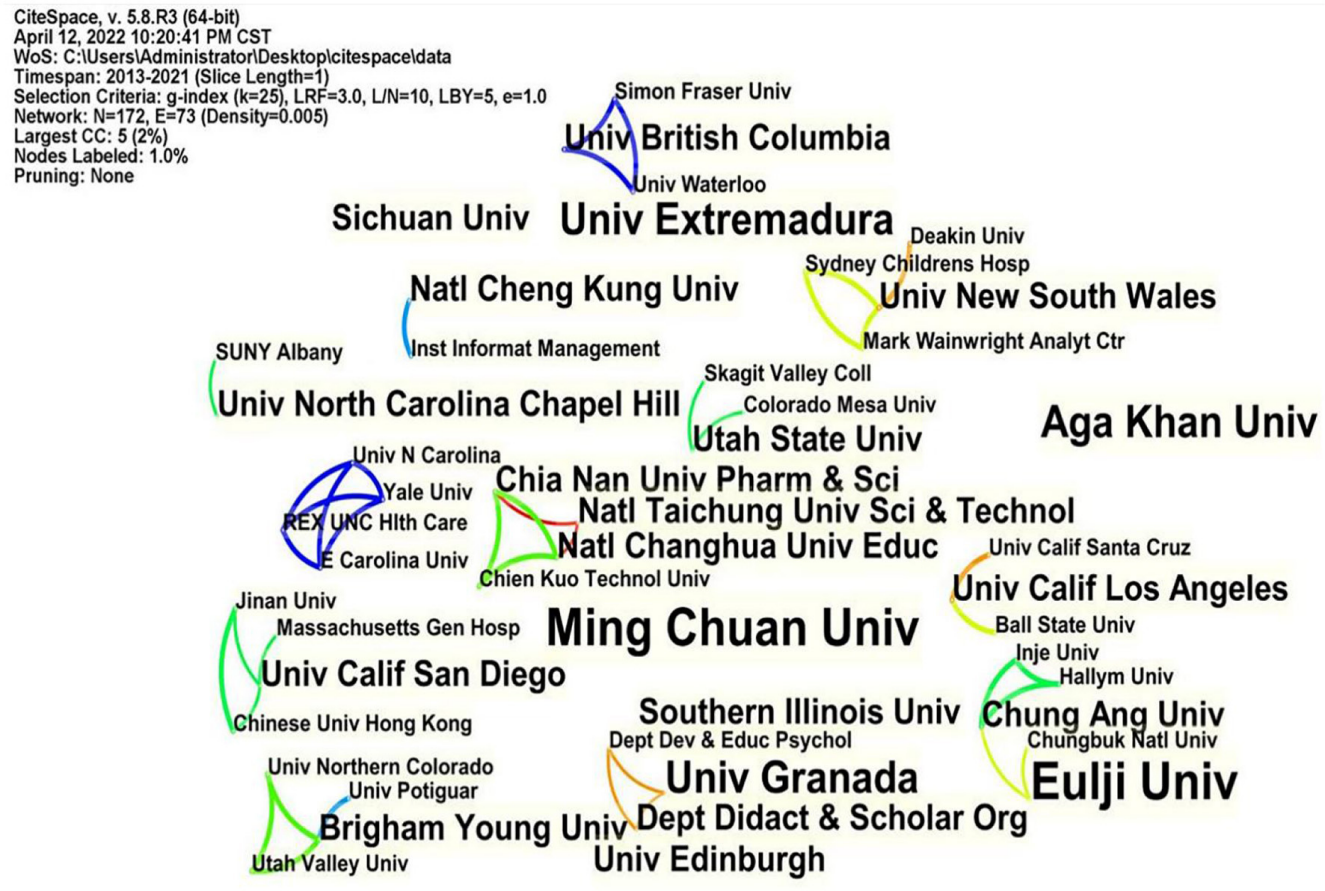
Research institutions mapping knowledge domains.

From the distribution of research institutions, it can be seen that the research institutions attach importance to this field. As seen from Figure 4, most research institutions are universities, indicating that flipped classrooms have attracted their attention. Ming Chuan Univ from Taiwan and Eulji Univ from South Korea published the most papers on flipped classrooms, with four pieces each. There were 73 links out of 172 research institutions, indicating a small number of clusters. Figure 4 shows that the cooperation between institutions is relatively sparse, lacking a coherent collaboration.

### National distribution

**Figure 5:**
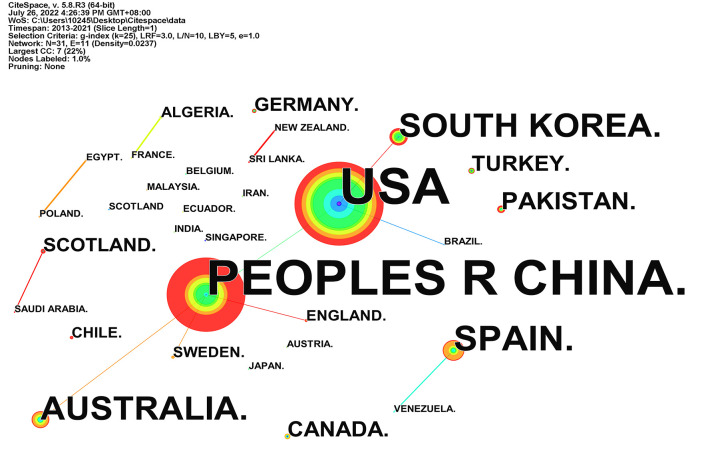
National distribution mapping knowledge domains.

Through the country distribution map, we can understand the international cooperation and the importance of different countries in this field. Table 2 lists the top five countries in terms of the number of publications, and these countries pay more attention to flipped classrooms. The United States tops the chart with 51 published papers accounting for 29.5%. Furthermore, the mediating centrality is 0.04, indicating that there is still a lack of highly cited articles. China ranks second with 46 reports, accounting for 26.3%. As shown in Figure 5 China has established the most cooperative relations with other countries, followed by the United States. Additionally, Spain, Australia, South Korea, and other countries also pay more attention to the flipped classrooms. The outcomes, *N* = 31, E = 11, indicate that the number of research countries in this field is relatively small. Figure 5 shows 31 countries with a global share of 13.3%. The number of countries studied was small and scattered, and most were independent, without forming a prominent cluster, and international cooperation needs to be strengthened.

**Table 2:** Country distribution and number of documents issued.

**Country**	**Centrality**	**Number of post**
USA	0.04	51
CHINA	0.09	46
SPAIN	0	13
AUSTRALIA	0	11
SOUTH KOREA	0	11

### Keyword cluster analysis

**Figure 6:**
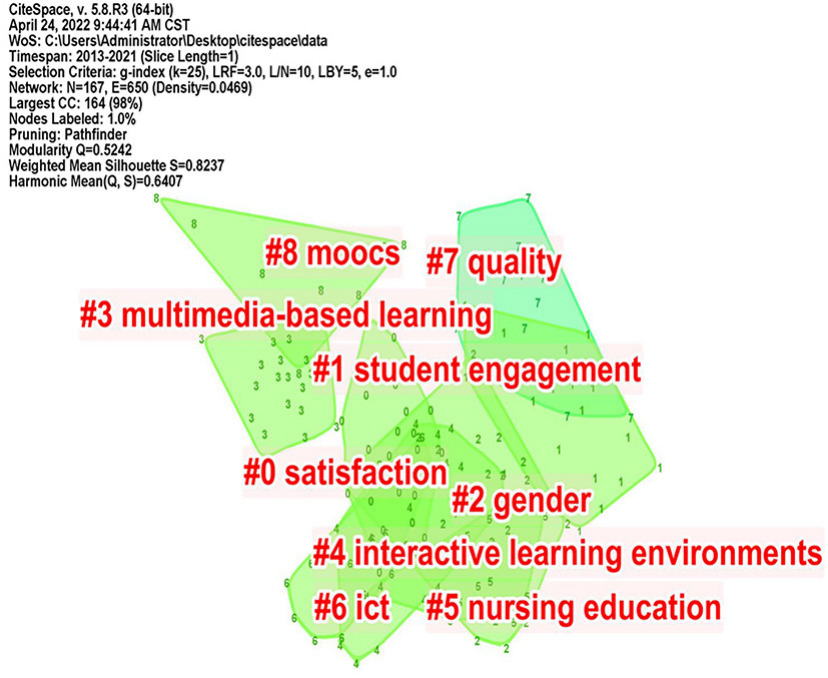
Keywords clustering mapping knowledge domains.

Keywords are highly concise summaries of the core content, which reflect the research value and direction of the article. High-frequency keywords are often used to determine hot topics and frontier evolution trends in a research field. The keyword cluster analysis was conducted using CiteSpace, as shown in Figure 6. Modularity Q represents the network's Modularity, Mean Silhouette represents the average contour value, and when Q>0.3 indicates that the clustering effect is significant when S>0.7 indicates high network homogeneity (48). Q = 0.5242, S = 0.8237 in the figure, shows that the clustering result has a significant effect, and there are nine clustering tags in total. Among them, the labels “#2 gender” and “#5 nursing education” appeared because many articles are about the application of flipped classroom in nursing education. However, the keywords in the clusters still conform to the hot topics of our analysis.

Based on the scientific knowledge map of keyword clustering, select “summary table | whitelists” in the “clusters” menu bar to get the high-frequency keywords clustering table, and select the top five keywords for each cluster (Table 3).

**Table 3:** High-frequency keywords clustering table.

**Cluster ID**	**Size**	**Silhouette**	**Top terms (take the first five)**
0	29	0.753	cooperative learning; learning strategies; improving classroom; satisfaction; redesign
1	22	0.856	student experience; checking; student engagement; active learning; reflective learning
2	22	0.856	motivation; digital divide; student performance; teaching strategies; improving classroom teaching
3	21	0.768	student-centered learning; self-instruction; multimedia-based learning; collaborative learning; cognitive load
4	20	0.905	interactive learning environments; computer-mediated communication; skills; learning communities; big data BOOCs platform
5	16	0.751	systematic review; design principles; application software education; achievement; accuracy
6	13	0.893	educational innovation; innovative methodologies; transformative pedagogies; student-centered learning; ICT
7	12	0.788	quality; blended learning; impact; knowledge; future teacher
8	9	0.921	distance learning; critical thinking; flipped classroom approach; MOOCs; evaluation

Through clustering analysis of high-frequency keywords in Figure 6; Table 3, the research content of flipped classrooms can be roughly summarized into the following three hot topics:

#### Topic 1: Preparation before class

Co-occurrence identifiers were learning strategies, teaching strategies, self-instruction, multimedia-based learning, cognitive load, big data BOOCs platform, design principles, application software education, educational innovation, ICT, distance learning, flipped classroom approach, MOOCs.

Pre-class activities require teachers to provide students with learning materials through the learning management system. Teachers use various software tools to create learning content, and then disseminate them to the student through video lectures, PowerPoint presentations, online tutorials, e-books, homework, and other forms (51–55). Students can study independently by watching the course content, or discuss and interact with teachers and classmates online (52, 55–57). The production of teaching videos is a challenge for teachers, as it requires making the video quality clear within a concise video length (58). Al-Zahrani mentioned the importance of the quality of the flipped classroom tools and materials, especially as video-recorded lectures and tools should be carefully prepared to increase student engagement and satisfaction (59). Low-quality video lectures may lead to poor learning outcomes (60). The video length should conform to the characteristics of students' physical and mental development, and be controlled within the time range of students' attention (61). The duration of the videos should range from 10 to 20 min (53). Each video should be targeted to a specific goal of the chapter, focusing on the perfect combination of classroom activities, and should be presented so that students can easily understand, thereby telling students what they need to know rather than simply directing them to read a specific chapter (62, 63). Videos released through the Internet have multiple features, such as pause and playback, which can be controlled by the students, and are convenient for review and consolidation. They are conducive to students' autonomous learning (64, 65). After watching the teaching video, students can judge whether they understand the learning content. The video is followed by homework, which can help students timely detect and judge their learning status (56). Assignments should cover the main points, but not be too extensive (66).

#### Topic 2: Classroom activities

Co-occurrence identifiers were cooperative learning, improving classroom, student engagement, active learning, reflective learning, motivation, student performance, student-centered learning, collaborative learning, interactive learning environments, skills, knowledge, learning communities, quality, blended learning, and critical thinking.

Constructivist learning theory advocates student-centered learning under the guidance of teachers. Teachers are the helpers and facilitators of the construction of meaning, while students process information, and are the active builders of meaning (67). In classroom activities, students change from passive listeners to active learners (68). Active learning is the main contribution of the flipped model and the most crucial factor affecting learning results (69). Teachers need to create specific teaching objectives and educational situations to stimulate students' interest in learning and assist students with learning motivation. Teachers should set class sizes, divide group discussion or teamwork, and conduct mixed teaching activities such as case studies, problem-solving, experiments, games, questions and answers, quotations, student presentations, inquiry learning, etc. (42, 66, 70). Classroom dialogue and discussions require careful preparation and observation by teachers to truly teach students according to their aptitude (51). Without the support of teachers and the help of peers, students often feel depressed, and lose their motivation and sense of achievement. The theory of interactive psychology emphasizes a great deal of peer interaction, and most teachers promote a high degree of interaction between group members in the form of task assignments (71). Teachers ask, guide, and answer questions through discussions among students, homework, and projects completed by students, and analyze and master the learning effect on students (68). Students can interact more effectively with the learning content while also increasing student-student interaction and student-teacher interaction (72–74). In flipped classrooms, students experience active learning and can engage in high-level thinking activities (75, 76).

#### Topic 3: Consolidate after class

Co-occurrence identifiers were satisfaction, redesign, student experience, checking, improving classroom teaching, computer-mediated communication, systematic review, achievement, accuracy, innovative methodologies, transformative pedagogies, impact, and evaluation.

In flipped classrooms, the consolidation stage after class is essential. Teachers need to pay attention to the evaluation and reflection of the classroom teaching effect, adjust corresponding teaching design schemes and teaching content based on students' learning interests and the problems encountered by students, and build a smooth information communication channel. In these classrooms, after-class discussions are mainly conducted online, the concepts or problems not been solved in the classroom can be discussed in a follow-up (55). After-class tests can evaluate students' learning, starting with more straightforward or more direct exercises and gradually increasing complexity over time, which helps cultivate mastery (77–79). After-class assignments should be diversified enough to help students summarize their skills and transfer them to other questions and courses (80). After-class evaluation is also an important part, for collecting feedback from participants, evaluating the effectiveness of flipped classrooms, and redesigning teaching strategies (52). Students can self-evaluate according to their learning attitude, confidence, and cooperation spirit in class, and then perform mutual evaluations with their peers, supplemented by their teacher's evaluation (79). Formative assessment tools can also be used to assess students' learning process in combination with a given set of standards, enabling learners to evaluate their performance through reflection and then modify it accordingly (81).

**Figure 7:**
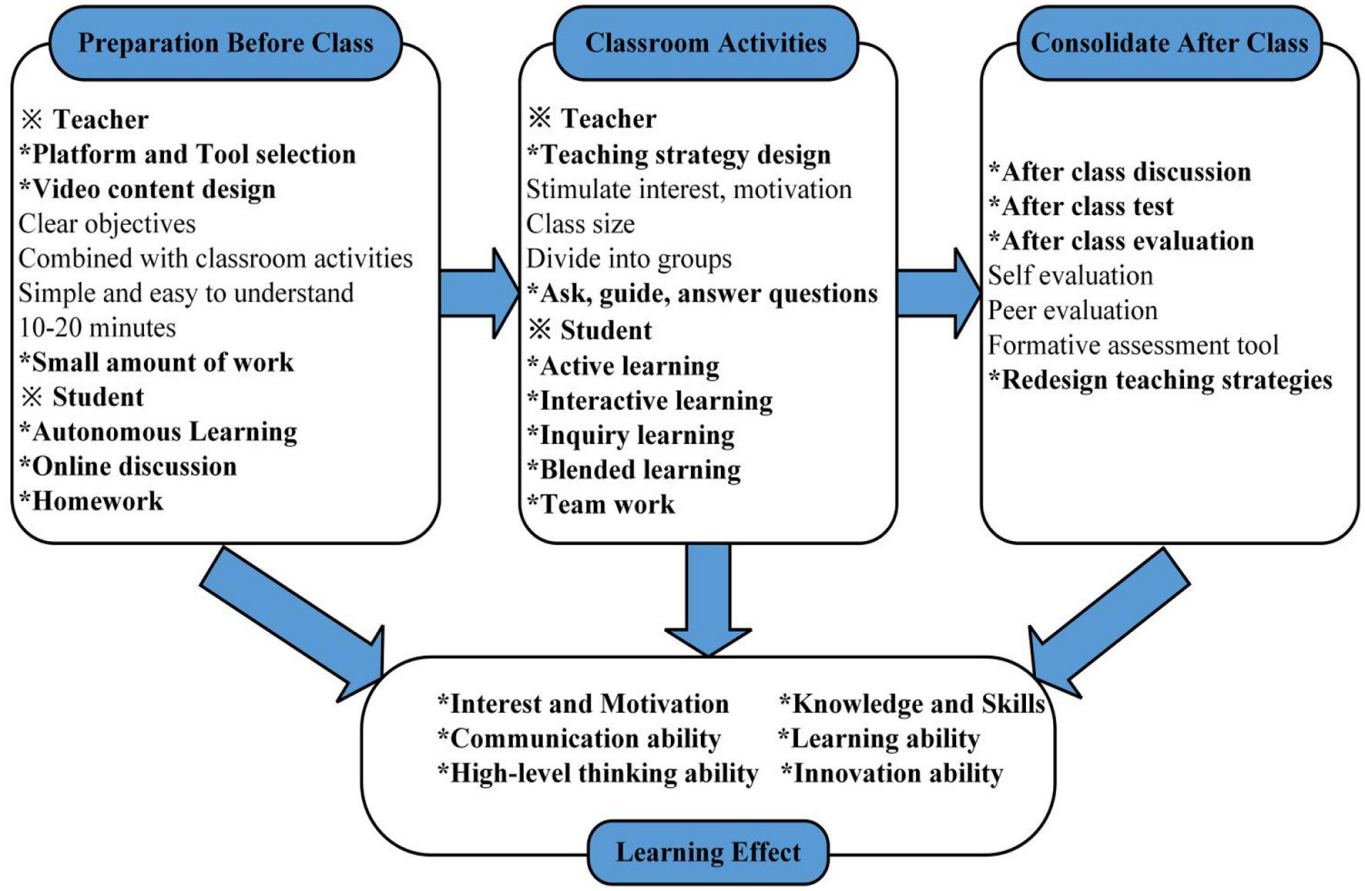
Flipping classroom teaching mode process.

By combing the hot topics of the flipped classrooms, the flow chart of flipped classroom teaching model is summarized as follows (Figure 7):

### Keywords time zone analysis

**Figure 8:**
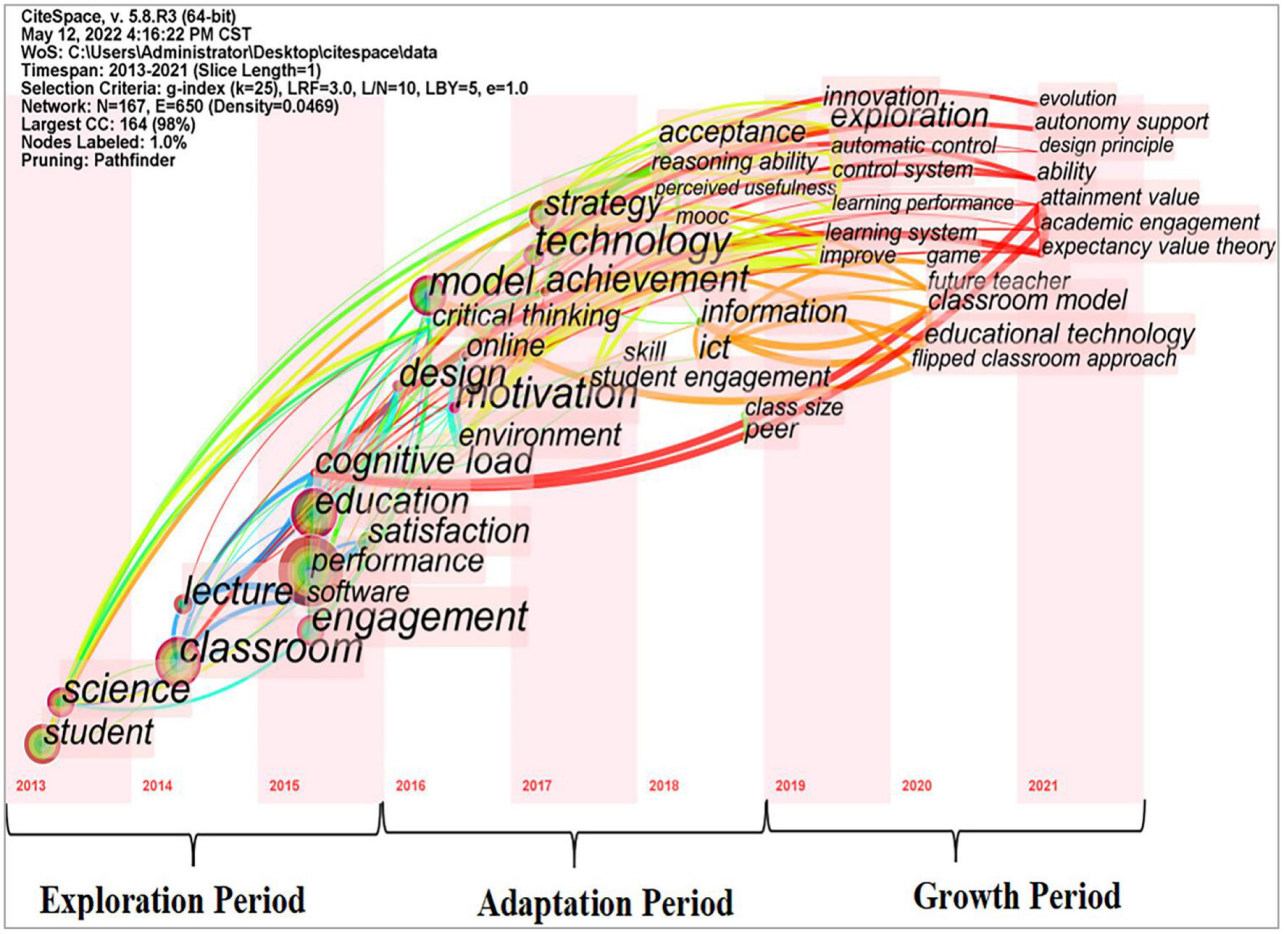
Keywords time zone map.

Through the identification and tracking of research frontiers regarding flipped classrooms, researchers can understand the research evolution dynamics at various stages of the discipline, predict the development trend of the research field, and identify problems that need to be further explored (48). According to the starting time of the emergence of keywords, the research frontier is divided into exploring period, adaptation period, and growth period to further clarify the research direction of the flipped classrooms in different periods (Figure 8).

#### Phase I: Exploration period

In this study, the years before 2015 was classified as exploration period, and the research frontier keywords of the flipped classrooms in this stage mainly focused on “student,” “classroom,” “lecture,” “software,” “engagement,” “performance,” and so on. It was a period of transition from traditional classroom lectures to flipped classrooms; The performance of teachers changed from active to passive, from being dominant to becoming the guides for students (52). This teaching mode required teachers to redesign their teaching strategies and thus required a significant amount of time investment in the initial implementation (51). The role of teachers had not been weakened but strengthened; which put forward higher requirements for teachers' professional quality (82). It was difficult for teachers who used the traditional education model to undergo such a transformation (66). For flipped classrooms, teachers should first create teaching videos with the help of scientific and technological tools, and may require time to redesign the course material in a flipped classroom (83). Some students have developed passive learning habits in the traditional classroom, which requires less time and work (60). Flipped classrooms need more time and work to preview learning materials, and students generally believe that extracurricular learning is an additional time burden (84). Low self-regulated behaviors by some students (85) may lead some students to schedule their time to comprehend the out-of-class learning content improperly (75). Some students were reluctant to accept it due to issues such as the limited Internet speed of students in rural or remote areas, the high costs of accessing high-speed Internet, and the delay and failure of accessing libraries and obtaining teaching videos, pictures, and audio files (86, 87). Therefore, in the early stage of implementing flipped classrooms, we should break the fixed teaching paradigm of teachers and students' learning habits and also consider the technical costs of the conversion to a flipped mode.

#### Phase II: Adaptation period

In this study, the period from 2016 to 2018 was divided into an adaptation period, and the keywords of the flipped classrooms in this stage were mainly concentrated on “online,” “motivation,” “critical thinking,” “technology,” “skill,” “strategy,” “ICT,” and so on. With the rapid development of information technology and the deepening of education reform, the reduction of information costs provides opportunities for updating university courses (88). The characteristics of information technology can better meet the needs of students for personalized teaching and improve the efficiency of the student-centered flipped classrooms (89). Once the course materials are developed, teachers need to spend less time to prepare for each lesson than before, because the instructional videos and related materials can be reused each year (74). Students quickly adapt, getting over their initial resistance and find the flipped classroom satisfying and effective (51). In this era of developing technology, students have access to various information and learning methods. In this period, problems caused by the technological gap have been reduced. The number of studies on flipped classroom teaching methods has steadily increased, probably due to the increasing popularity of Internet technology (60). The research results of the flipped classrooms during this period show that students have achieved good learning results in many aspects, such as learning attitude and motivation, practical ability, critical thinking ability and caring ability, sense of self-efficacy, learning interest and autonomous learning ability, self-regulation and cooperation, high-order thinking, independent and creative thinking, perceived usefulness, academic achievement and teamwork, insight, challenge, relevance and responsibility, knowledge and skills (75, 90–100).

#### Phase III: Growth period

This study divided the period from 2019 to 2021 as a growth period, and the keywords in this stage were mainly concentrated on “control system,” “automatic control,” “learning system,” “educational technology,” “classroom model,” “game,” and so on. Due to the emergence of big data, the Internet of Things, and artificial intelligence education, lecture content produced by digital technology, as well as audio and video related to teaching knowledge, provided technical support for the implementation of flipped classrooms and flipped classrooms had become a teaching strategy commonly used in universities (101–103). As a result of the outbreak of the COVID-19, the traditional university courses have changed dramatically from face-to-face teaching to online teaching (104). Online tools and resources, such as MOOCs, ZOOM, Learning Management System (LMS), etc., have been widely adopted by universities (105, 106). These learning platforms not only support multiple application scenarios, but an interactive teaching experience is also provided (70). With the emergence of digital video recording, digital media, laptops, and smartphones, it is easier to integrate the flipped classroom teaching model into online teaching. Hybrid learning methods (face-to-face teaching, online teaching, teamwork, etc.) enrich the flipped classroom teaching method (78, 107). Teachers utilize modern information technology to their advantage and compile content and knowledge points into vivid and convenient micro-videos. Students tend to use emerging technologies as tools to acquire new knowledge (108). Digital experience has become a part of the learning habits of the new generation of students (109).

## Discussion

The advent of the era of big data has provided favorable conditions for the development of flipped classrooms. The number of studies on flipped classrooms has also increased yearly. Most studies show that the flipping teaching mode improves students' academic performance, and students generally prefer the flipping mode rather than the traditional teaching model. However, there is no united conclusion regarding the effect of this teaching model, and more research is needed to test the advantages of this model. Combined with the analysis of existing literature, this study discusses the following aspects to provide a reference for follow-up research.

### Mutual cooperation

At present, there are few studies on the flipped classroom teaching model, few strong cooperation teams between authors, the various research institutions do not form a close network, the regional development is uneven, and the international differences are apparent. Only a few countries, such as the United States, China, Spain, Australia, South Korea, England, Scotland, and Brazil, etc. have a partial cooperation, and many countries do not pay enough attention to the flipped classroom. Although flipped classroom teaching has brought many advantages, it is not as popular in many countries and regions. On the one hand, flipped classroom may not have formed a mature theoretical system and needs to be tested in a variety of professional disciplines. Relevant research scholars need to strengthen contact and cooperation, explore systematic teaching methods suitable for different fields, and conduct practical testing and evaluation. On the other hand, it may be due to the limitations of educational resources, technical equipment, and other conditions. Educational institutions should establish high-quality video resource databases and advanced information technology platforms, strengthen international cooperation and realize the sharing of educational resources, which is of great significance to promote the balanced development of education and promote the flipped teaching model.

### Teachers and students

The development of modern education to the stage of Constructivism education recognizes the role of students in learning. However, some scholars have pointed out that some students are not good at asking questions and are weak in taking initiative. Some students lack the ability for independent learning and cooperative learning (110, 111). These phenomena directly affect the effect of the flipped classrooms. Through literature review, we discovered the following reasons: First, each learner has different acceptance of cognitive load. The presentation of varying teaching contents and the complexity of tasks may lead to differences in cognitive load, and affect the process of learners absorbing knowledge. Additional learning materials may lead to problems in students' understanding of different levels (112). Therefore, the unnecessary cognitive load should be reduced in the teaching design. Second, teachers' teaching strategy, video quality, and educational concept also affect the teaching effect (101, 113). It is necessary for teachers to provide personalized guidance according to the characteristics of each learner, put forward appropriate questions to guide students' thinking and discussion, and lead the problems step by step to deepen students' understanding of the learned content; Teachers should inspire and induce students to discover the law, correct and supplement their wrong and one-sided experience. Furthermore, in the era of information explosion, teachers need to be proficient in subject knowledge, and need to improve information literacy. Teachers can choose digital tools suitable for teaching methods according to the difference between digital tools and software proficiency using information technology to make teaching videos. The effective utilization of digital teaching resources is an essential ability for every educator, which can effectively promote the reform of flipped classrooms and improve teaching quality. Third, face-to-face interactive learning activities are the most essential value of flipped classrooms, which help to enhance students' academic performance. As the basic theory of interpersonal interaction, interactive psychology can deal with various problems, such as parent-child interaction, husband and wife interaction, teacher-student interaction, social interaction, and natural interaction. Based on the theory of interactive psychology, students should pay attention to psychological literacy. Teachers can understand students' psychological state through students' performance, provide students with relevant psychological guidance and improve students' interactive ability.

### Online flipped classroom

With the outbreak of COVID-19, university teaching has shifted from offline to online. Compared with face-to-face instruction, online education lacks adequate supervision, communication, interaction, and cooperation, and it is difficult for teachers to grasp the actual situation in these classrooms. Garcia-vedrenneae et al. found that the transition from flipped classroom to online teaching can be simplified by communicating with students, flexibly arranging curriculum structure, and assessing specific needs (114). Flipping learning is increasingly used in online education in many countries (42). The well-designed online flipped teaching offers teachers another way to maintain quality education when crises break out (115). The flipped teaching can improve students' motivation in distance learning (116). Through the e-learning platform such as the Massive Open Online Courses (MOOC), and the Tencent Classroom, online courses can be effectively integrated into the flipped teaching mode and effectively mobilize students' active participation, communication, and interaction (117). First, to ensure the teaching quality of online flipped classrooms teachers should set appropriate course task difficulties. When the task is difficult, the value of interest may hinder rather than promote students' behavioral participation. Second, before each course task, teachers should reasonably divide students into groups and improve students' involvement through teamwork, question and answer, raising hand keys and interaction in the information bar. Third, teachers should establish an incentive mechanism to promote group interaction. At the end of each semester, students with best performance in the group should be rewarded. Finally, the objective and multi-dimensional evaluation through digital means is conducive to teachers' fundamental understanding of students' learning knowledge. The follow-up of evaluation technology can effectively improve the teaching quality and promote the popularization of the online flipped classroom.

### Future flipped classroom

Under the tide of the digital transformation of education, flipped learning has become an important trend in education in recent years. Educational informatization is also facing the shift from Information Technology to Data Technology. Colleges and universities have developed from only focusing on micro-courses in the initial stage, to reshaping and reengineering the teaching business process, and then to the systematic reform of school education. Big data will open a new era of comprehensive and in-depth integration of artificial intelligence and education (118). The flipped classroom addresses the teaching needs created in the context of a global health emergency, while using digital tools to ensure social distancing and connection between teachers and students (119). Artificial intelligence education and machine learning allow teachers to capture and publish online course content, which students can easily access after class. With the help of teachers, students realize their growth. Students independently plan learning content, rhythm, style, and presenting knowledge (120). Independent development will replace passive education. In the future, the school will build an intelligent education ecosystem integrating the Internet of Things, Digital Alliance and Zhilian, focusing on cultivating students' core literacy. The future learning and education environment will continue to improve. Each teacher has an artificial intelligence assistant to support personalized learning for each student. The high utilization rate of students' networks and the emergence of interactive web pages will help promote the digitization of the flipped classroom teaching model. Education authorities must strengthen the technical tools and teacher skills training needed to develop the capacity to respond quickly to current and future teaching challenges (119).

## Conclusion

This study uses the scientific measurement software CiteSpace to draw a scientific knowledge map of 175 studies related to flipped classrooms in the WOS database. It performs a visual analysis of the research author who published relevant papers, research institution, country, keyword clustering, keyword co-occurrence and keyword time zone distribution, to systematically determine the research status, hot topics and cutting-edge evolution trend of flipped classroom teaching mode. The results show that: first, although the research on the flipped classrooms shows a growing trend, the overall number of research is not high. Relevant authors have conducted comparative research on flipped classrooms and traditional classrooms in many disciplines, but no unified research conclusions have been reached. Some flipped classrooms improve learning interest, some improve satisfaction, and some students like the conventional model. So far, a flipped teaching model suitable for different disciplines has not been formed. The number of countries studying flipped classrooms is generally small. Only a few countries, such as the United States, China, Australia, Spain, South Korea, and so on, pay great attention. In the future, it is necessary to strengthen the academic cooperation among authors across institutions or countries to share high-quality resources and jointly improve teaching quality. Secondly, with the development of big data, the Internet and artificial intelligence, the flipped teaching model has gradually become one of the directions of teaching reform. Students will give full play to the central role of learning. Students' initiative, interaction, peer cooperation and teamwork in flipped classrooms play an essential role in education. Simultaneously, teachers should have higher professional and information literacy. They should make high-quality teaching videos by using information technology, and design teaching programs with clear objectives and deepen them layer by layer to stimulate students' learning interest and participation, and carry out practical scientific evaluation of the flipping effect, to adjust the design program constantly. Finally, relevant literature shows the feasibility of the online flipping mode, which can effectively make up for the shortcomings of online teaching. Moreover, with the advent of digital intelligence, the digitization of flipped classrooms will bring higher quality teaching resources and environments.

## Research significance

Through combing the relevant literature on the flipped classrooms in colleges and universities, we can see that the online flipped teaching model can be effectively utilized in the context of the COVID-19 pandemic. This online version has important theoretical significance and practical value for the innovative flipped education model. From a theoretical point of view, this study reveals the current implementation status and future trend of the flipped classroom education. It shows the focus of the researchers on the perceived effectiveness of this teaching model and the research direction in this field, which lays a theoretical foundation for future academic research. From a practical point of view, this study emphasizes the role changes of teachers and students in the flipped classrooms. Teachers can use rich teaching videos to provide students with multi-directional learning resources, and students can flexibly choose the time of video learning and interesting information. The interaction time between teachers and students is increased in the classroom, and the classroom efficiency is greatly improved, which helps to cultivate students' thinking, communication and cooperation ability. During the pandemic, the online flipping teaching can fully mobilize students' learning enthusiasm and enrich students' knowledge and skills. This research can provide a meaningful reference point for the practical implementation of flipping classrooms.

## Limitations

The main limitation of this study was that it only selected the papers written in English on flipped classroom teaching methods from the web of science database and did not select the papers in other databases and other languages, which may lead to a lack of information. Moreover, the choice of literature related to the subject was influenced by human subjectivity. These limitations can be further addressed in future research.

## Data availability statement

The datasets presented in this study can be found in online repositories. The names of the repository/repositories and accession number(s) can be found in the article/supplementary material.

## Author contributions

LY designed and conceptualized the study and wrote the manuscript. LS supervised the project, obtained funding, and reviewed and edited the manuscript. XW participated in screening the articles and provided critical feedback. JZ and XZ were responsible for software and validation. All authors contributed to the article and approved the submitted version.

## Funding

This study was funded as part of a major project in Humanities and Social Science Research of Higher Education Institutions in Anhui Province (SK2021ZD0039), Science and Technology Innovation Strategy and Soft Science project of Anhui Province (202106f01050043), and Social Science Innovation and Development Research Project of Anhui Province (2021CX508).

## Conflict of interest

The authors declare that the research was conducted in the absence of any commercial or financial relationships that could be construed as a potential conflict of interest.

## Publisher's note

All claims expressed in this article are solely those of the authors and do not necessarily represent those of their affiliated organizations, or those of the publisher, the editors and the reviewers. Any product that may be evaluated in this article, or claim that may be made by its manufacturer, is not guaranteed or endorsed by the publisher.
